# The role of reassurance seeking in obsessive compulsive disorder: the associations between reassurance seeking, dysfunctional beliefs, negative emotions, and obsessive- compulsive symptoms

**DOI:** 10.1186/s12888-020-02766-y

**Published:** 2020-07-07

**Authors:** Bikem Haciomeroglu

**Affiliations:** Department of Psychology, Ankara Hacı Bayram Veli University, Emniyet Mahallesi Abant 1 Cad. No:10/2D Yenimahalle, Ankara, Turkey

**Keywords:** Reassurance seeking, Obsessive compulsive symptoms, Dysfunctional beliefs, Emotions

## Abstract

**Background:**

This study investigates the association of reassurance seeking with obsessive compulsive (OC) symptoms, dysfunctional beliefs, and negative emotions.

**Methods:**

Reassurance Seeking Questionnaire, Obsessive-Compulsive Inventory, Obsessive Beliefs Questionnaire, Trait Anger Expression Inventory, and Guilt Inventory were applied to 53 obsessive compulsive disorder (OCD) patients and 591 non-clinical participants.

**Results:**

The results showed that the severity of the OC symptoms significantly predicted the carefulness of OCD patients during reassurance seeking, indicating increased carefulness during reassurance seeking as the severity of OC symptoms increased. Moreover, feelings of guilt increased with increasing intensity of reassurance seeking. In addition, carefulness during reassurance seeking significantly predicted the level of anxiety. Responsibility/threat estimation, perfectionism/need for certainty, and importance/control of thoughts significantly predicted the OC symptoms. Moreover, the dysfunctional beliefs directly associated with an increased need to seek reassurance from different sources and seek reassurance more carefully. In terms of mediational effect, the results revealed that the individuals who had distorted beliefs were more likely to have OC symptoms and, in turn, the OC symptoms increased carefulness during reassurance seeking. The analysis of the model test revealed mostly similar results to those obtained for the clinical sample.

**Conclusions:**

The findings revealed a close relationship between OC symptoms and reassurance-seeking behaviors.

## Background

Reassurance seeking in Obsessive Compulsive Disorder (OCD) has been conceptualized as a form of neutralization behavior. The anxiety of a person is decreased by seeking reassurance, since it reduces the perceived threat, perceived probability of occurrence of a feared event, and perceived responsibility for negative consequences [32]. Salkovskis’ [37] description of reassurance seeking as an attempt to “put things right” points out how reassurance seeking functions. Reassurance seeking not only provides confirmation that the person took the necessary precautions to prevent harm but also passes some of the responsibility to the person offering reassurance [37, 38]. However, similar to other OCD-related neutralization and safety behaviors (e.g., checking, washing, thought suppression, distraction, and mental ritualization), anxiety reduction by obtaining reassurance is temporary [14, 32, 34, 39]. In the long term, it prevents the disconfirmation of the feared consequences and contributes to the maintenance of OC symptoms.

Previous studies have shown the associations between the severity of obsessive-compulsive symptoms and reassurance-seeking behaviors. Starcevic et al. [45] found that individuals who used reassurance seeking had more severe obsessions than the individuals who did not, suggesting that the severity of the obsessions can lead the individuals to use coping strategies such as reassurance seeking in addition to other types of compulsions. In terms of the associations between symptom subtype and reassurance seeking, many studies provided empirical evidence for the co-occurrence of compulsive checking and reassurance seeking [28, 32, 35, 36, 44, 45]. From a cognitive perspective, Salkovskis [37, 38] and Rachman [32] pointed out the similarities between reassurance seeking and checking. Thus, the researchers proposed conceptualizing reassurance seeking as a special type of checking behavior. Although Kobori, Salkovskis, Read, Lounes, and Wong [16] acknowledged that reassurance seeking is particularly common among individuals with checking compulsions, they emphasized the importance of studying the relationship between reassurance seeking and other types of OCD symptoms as well. The number of studies showing the associations between reassurance seeking and obsessions with cleaning, aggression, sexual, somatic, and religious contents is relatively low [1, 16, 53].

Despite the proposed similarities and co-occurrence of reassurance seeking and checking behaviors, the “interpersonal aspect” of reassurance-seeking behaviors prompted researchers to provide a stronger justification for studying reassurance seeking as a separate construct or at least as a sufficiently unique behavior within a broader domain of checking. In this scope, distinguishing “self-reassurance” from “reassurance seeking from others” can be useful to understand the concept of reassurance seeking as it is discussed in this study. In self-reassurance, the OCD patients use mental checking and self-talk to reassure themselves (such as, to keep telling oneself that “the door is locked”), especially if there is no one to serve as a source of reassurance or they feel negative emotions such as embarrassment if they ask for reassurance [15]. Therefore, self-reassurance, a kind of mental checking, can have similarities to behavioral checking as both are carried out by the individual him/herself. However, the “interpersonal aspect” is the important characteristic of reassurance seeking from others, which distinguishes it from self-reassurance and checking behaviors [15, 16]. The interpersonal aspect can be explained by the OCD patients’ need for “others” to give them reassurance and to reduce the anxiety caused by obsessions. Different from other neutralization strategies, the OCD patient seeking reassurance as a safety strategy, for example, not only checks the door several times to make sure it is locked but also requires a trusted or loved one to accompany the person in different ways, i.e. watch him/her while checking, verbally reassure him/her, or join in with his/her ritual. By this way, the reassurance provider, intentionally or unintentionally, verifies his/her awareness of the situation and precautions taken to prevent possible negative consequences. Moreover, the “interpersonal aspect” also manifests itself as emotional distress both in the OCD patient and reassurance provider during seeking and providing reassurance, which usually creates problems in their relationship.

Cognitive-behavioral models of OCD have emphasized several cognitive beliefs and dysfunctional attitudes such as inflated responsibility, overestimation of threat, intolerance of uncertainty, perfectionism, importance of thoughts and importance of controlling the thoughts [8, 24, 31, 32, 37, 38]. However, the specificity of the dysfunctional beliefs to OCD has been debated by many researchers. In their comprehensive meta-analysis, Pozza and Dettore [29] found a medium effect size on the relation of responsibility beliefs to OCD symptoms. Although responsibility beliefs were found to be more strongly associated with OCD than depression symptoms, they are equally associated with OCD and anxiety symptoms with a stronger relation to OCD at a trend level [29]. Moreover, due to the heterogeneous symptom profile of OCD, certain obsessive beliefs were found to be more associated with certain OCD symptom dimensions, i.e. contamination symptoms and responsibility/threat estimation beliefs, symmetry symptoms, and perfectionism/certainty beliefs, etc. [50].

A limited number of studies examining the specific associations of dysfunctional beliefs with reassurance seeking have mainly focused on inflated responsibility attitudes. Salkovskis et al. [40] stated that an inflated sense of perceived responsibility increases not only the occurrence of obsessions but also the use of counter-productive neutralization strategies such as reassurance seeking. In an experimental study [27], participants under a high responsibility/threat condition expressed more need to check and seek reassurance than the ones under a low responsibility/threat condition. Although inflated responsibility and threat appraisals seem to be the major motivations to seek reassurance, in their qualitative study, Kobori, Salkovskis, Read, Lounes, and Wong [16] pointed out the role of other dysfunctional beliefs. The researchers stated that other motivations to require reassurance involved obtaining feelings of perfection, certainty, and rightness.

Although there have been many empirical studies supporting the role of belief domains in the development and maintenance of OCD, some of the variances in the severity of OCD symptoms remains unaccounted for. Unexplained variance leads researchers to examine other factors to obtain a more comprehensive understanding of OCD. Therefore, the examination of emotional responses is important in the explanation of the heterogeneous nature of OCD in addition to the cognitive appraisals [42]. Anxiety obviously plays a central role in OCD; however, other aversive emotions play an important role in the etiology of OCD as well. Consistently, the research has indicated a positive association between the severity of OCD symptoms and emotions such as disgust [4, 21, 33], shame/guilt [20, 30, 41], and anger [51, 52].

The aversive emotions experienced by OCD patients are possibly not only related to the obsession itself, which has a content mostly contradicting the self, but also to the neutralizing behaviors. There is a relatively small number of empirical studies investigating the negative emotions emerged by neutralizing behaviors, specifically by reassurance-seeking behaviors. Kobori, Salkovskis, Read, Lounes, and Wong [16] determined that individuals with OCD had a strong motivation to seek reassurance, but they simultaneously had concerns about the consequences of reassurance seeking. In their qualitative study, they found that the concerns of the individuals mainly focused on the possible negative impacts of reassurance seeking on the reassurance provider. Therefore, the individuals who used reassurance seeking also reported indecision, conflicting emotions, and inhibitions during reassurance seeking. Kobori, Salkovskis, Read, Lounes, and Wong [16] stated that asking for reassurance resulted in feelings of embarrassment and fear, since the person knew that he/she annoyed or exhausted the person who provided reassurance. Similarly, Parrish and Radomsky [28] found that interpersonal concerns (such as fear of embarrassment or making the reassurance provider angry/frustrated) were the factors that lead participants to stop seeking reassurance. In another study examining the feelings of the participants when they received or failed to receive reassurance, the results indicated that reassurance reduced anxiety and increased the urge to seek it more. Individuals with OCD reported that they felt a greater urge to seek reassurance when the reassurance was not provided [17]. In their study, Halldorsson and Salkovskis [12] found that excessive reassurance seeking commonly created relationship problems and feelings of frustration.

### Aims of the study

This study examines the cognitive, emotional, and behavioral factors associated with the symptoms of OCD in a comprehensive model. More specifically, the study focuses on the associations of dysfunctional beliefs, negative emotions, and reassurance-seeking behaviors with OCD symptoms. Various empirical studies have previously verified the role of dysfunctional beliefs and negative emotions in OCD; however, the present study examines the specific role of reassurance-seeking behaviors in OCD. Based on the Salkovskis’ [37, 38] model of OCD, in this study, reassurance seeking was accepted as a neutralization and safety behavior which reduces the anxiety and distress caused by obsessions. According to this model, interpretation of the occurrence and/or content of the intrusion are influenced by pre-existing dysfunctional beliefs. This type of interpretation leads the individual to engage in overt and/or covert neutralizing behaviors, reassurance-seeking behaviors, and negative mood changes. In other words, pre-existing dysfunctional beliefs make the person more prone to appraise the intrusions as threatening and uncontrollable. Therefore, the proposed model tests the mediator role of OCD symptoms in the relationship between dysfunctional beliefs and reassurance-seeking behaviors. Different models can also be proposed to explain the associations between the symptoms of OCD and dysfunctional beliefs, negative emotions, and reassurance-seeking behaviors, since reassurance-seeking behaviors also contribute to the increment of OC symptoms in the long term, leading to a vicious cycle in which OC symptoms become an outcome of the reassurance-seeking behaviors. Although there are a considerable amount of studies showing the specific associations between dysfunctional beliefs, OC symptoms, and negative emotions, the number of studies on reassurance seeking in OCD is very limited. Therefore, this study has an explanatory nature that explores the associations between previously studied factors (OC symptoms, beliefs, and emotions) and reassurance-seeking behaviors. It was hypothesized that a) there are significant associations between reassurance-seeking behaviors and OC symptoms, b) there are significant associations between reassurance-seeking behaviors and negative emotions c) there are significant associations between reassurance-seeking behaviors and dysfunctional beliefs, d) OC symptoms mediate the relationship between dysfunctional beliefs and reassurance-seeking behaviors, and, finally, e) OC symptoms and reassurance-seeking behavior mediate the relationship between dysfunctional beliefs and negative emotions. All hypotheses were examined using the OCD sample, except for the final proposed model which was analyzed using the non-clinical sample due to the small sample size of the OCD group.

## Method

### Participants

The clinical sample consisted of OCD patients (*N* = 53). The non-clinical group consisted of university students and staff (*N* = 591). The OCD group included 22 females and 31 males with a mean age of 31.45 (SD = 10.66). Individuals who applied to the Psychiatry Department of an education and research hospital were assessed by trained psychiatrists using the Structured Clinical Interview for DSM-IV Axis I Disorders (SCID-I [9, 26];). No patient was excluded due to not meeting the diagnosis of OCD. Patients diagnosed with OCD were informed about the study by the researchers and patients voluntarily participated in the study. Seventeen subjects with a comorbid diagnosis (i.e. substance dependency, bipolar disorder, depression, etc.) were excluded from the study. Five (9.4%) participants had a postgraduate degree, 20 (37.7%) participants had a graduate degree, 20 (37.7%) participants had a high school degree, and 8 (15.1%) participants were primary or secondary school graduates.

The non-clinical sample consisted of 481 university students and 110 university staff (468 females and 120 males with a mean age of 22.74, SD = 6.94). Thirty-six (6.1%) participants had a postgraduate degree, 529 (89.5%) participants were undergraduate students, 19 (3.2%) participants had a high school degree, and 4 (0.6%) participants were primary or secondary school graduates. In the non-clinical sample, 41 subjects who reported having been diagnosed with a psychiatric disorder and/or going through treatment in the demographic information form were excluded from the study.

### Measures

#### Obsessive-compulsive inventory-revised form (OCI-R)

Developed by Foa et al. [10], the OCI-R is an 18-item self-report measure of obsessive-compulsive symptoms. The OCI-R consists of six subscales: Checking, Neutralizing, Obsessing, Hoarding, Ordering, and Washing. The responses are rated on a 5-point Likert-type scale. The total OCI-R score is obtained by summing all items. The coefficient alphas were .86 for Washing, .88 for Checking, .90 for Ordering, .82 for Obsessing, .90 for Hoarding, .86 for Neutralizing, and .81 for total score in the OC group. The test-retest reliability in the OC group ranged from .74 to .91. Significant positive correlations were found between the OCI–R total score and other OCD measures [10]. The OCI-R was translated into Turkish by Yorulmaz, Inozu, Clark, and Radomsky [55]. Confirmatory factor analysis supported the factorial structure of the original OCI-R (CFI = 0.92, GFI = 0.90, RMR = 0.05). The Turkish OCI-R also had internal consistency with α coefficients ranging from .64 to .80 for the subscales and .84 for the total score. In the present study, alpha coefficient for the total score was .89 both for the OCD and non-clinical groups.

#### Obsessive beliefs questionnaire (OBQ)

The OBQ-44 is a self-report measure that evaluates various maladaptive beliefs characterizing OCD [22–24]. The measure consists of three subscales: Responsibility/Threat Estimation, Importance/ Control of Thoughts, and Perfectionism/Certainty. The scale was adapted into Turkish by Yorulmaz and Gençöz [54]. Three factor structure was confirmed for the Turkish version having internal consistency with alpha coefficients of .86 for Perfectionism/Certainty, .80 for Importance/ Control of Thoughts, .85 for Responsibility/Threat Estimation, and .92 for total score. In the present study, alpha coefficient for the total score was .93 for the OCD group and .94 for the non-clinical group.

#### Reassurance seeking questionnaire (ReSQ)

The ReSQ [15] measures the range of manifestations of reassurance-seeking behaviors. The Likert-type scale has four subscales comprising the Source, Trust, Intensity, and Carefulness subscales. Source scale assesses how frequently participants seek reassurance from a range of sources (family members, close friends, strangers, health professionals, technical professionals, religious authority, books, websites, and self-reassurance). Twenty-one items were scored on a scale of never (0), rarely (1), sometimes (2), often (3), very often (4), and always (5). Trust scale assesses how much participants trust a range of information sources. Sixteen items were scored on a scale of not at all (0), slightly (1), partially (2), moderately (3), strongly (4), and completely (5). Intensity scale measures how many times participants seek the same reassurance. Sixteen items were scored on a scale of never (0), only once (1), twice or three times (2), four to six times (3), and many times (4). Carefulness scale measures how frequently participants are careful when they seek reassurance. Eleven items were scored on a scale of never (0), rarely (1), sometimes (2), often (3), very often (4), and always. The questionnaire was adapted to Turkish by Haciomeroglu and Inozu [11]. Confirmatory factor analysis supported the factorial structure of the original ReSQ. The internal consistencies were .89 for Source, .89 for Trust, .90 for Intensity, and .81 for Carefulness. The test-retest reliabilities were .67 for Source, .61 for Trust, .66 for Intensity, and .72 for Carefulness. The scale has also satisfactory convergent, discriminant, and criterion validity. For the present study, alpha coefficients were .88 and .89 for Source, .89 and .87 for Trust, .89 and .88 for Intensity, .81 and .85 for Carefulness, respectively, for the OCD and non-clinical groups.

#### State and trait anger expression inventory (STAXI)

The STAXI is a 4-point Likert-type scale developed to measure expressed anger levels [43]. The first 10 items of the scale measure trait anger, while other 24 items measure the anger expression styles of individuals (i.e., anger-in, anger-out, and anger control). The scale was adapted to Turkish by Özer [25]. The internal consistency coefficients of the scale were found to be .79 for Trait Anger dimension, .84 for Anger Control, .78 for Anger-Out, and .62 for Anger Suppression [25]. In the present study, alpha coefficient for the total score was .82 for the OCD group and .79 for the non-clinical group.

#### Guilt inventory (GI)

The GI [18] consists of 45 items that assess trait and state guilt as well as moral standards. Items are scored on a five-point scale (0 = very strongly disagree; 4 = very strongly agree). The inventory was adapted to Turkish by Akin, Haciomeroglu, and Inozu [2]. The reliability and validity coefficients were comparable to the original study. The internal consistency for the total score was .91 and the four-week test-retest correlation was .83 (*n* = 164, *p* < .01). In the present study, alpha coefficient for the total score was .85 for the OCD group and .79 for the non-clinical group.

### Procedure

The OCD group was asked to fill out a booklet comprising the Informed Consent form, Demographic Information Form, OBQ, OCI-R, ReSQ, GI, and STAXI after assessment using the SCID-I. The instruments were applied to university students during regular class hours after taking the consents of the students and their instructors. The questionnaires were applied to voluntary university staff whenever they were available. Before the administration, all participants were informed about the aims of the study. The questionnaires were applied in a randomized fashion to avoid the sequencing effect. The application took about 40–50 min. Ethical approvals were obtained from the related ethics commissions.

### Statistical analyses

Before the main analyses, the data was checked for missing values, outliers, and distributional properties towards meeting the relevant assumptions for the planned statistical tests. Prior to analyses, missing data (< 1%) in the questionnaires were replaced with the participant’s mean score on the respective measure [47]. All measures were screened for normality, distributions of all continuous data were checked, and the results were found to be satisfactory. All research questions were examined in the OCD sample, except for the final proposed model which was analyzed using the non-clinical sample due to the small sample size of the OCD group. Multiple regression analyses were conducted using the OCD sample to investigate the associations between reassurance seeking and OC symptoms, dysfunctional beliefs, and negative emotions. Then, linear regression analyses were chosen to test the mediator role of OC symptoms in the relationship between dysfunctional beliefs and reassurance-seeking behaviors in the OCD sample. The PROCESS, a computational tool available for SPSS, was used to test the indirect effects (i.e., mediation) [13]. Finally, Structural Equation Modeling (SEM) was conducted using the Analysis of Moment Structures (AMOS) in the non-clinical sample to test the model, which was proposed to clarify how all variables were related to each other.

## Results

### Descriptive statistics for participants’ scores on the scales

Before the main results, means and standard deviations of the scales were presented in Table 1.

**Table 1:** Descriptive statistics for participants’ scores on the scales

	OCD group (*N* = 53) Mean (SD)	Non-clinical control group (*N* = 591) Mean (SD)
OCI-R	2.18 (.75)	1.40 (.69)
OBQ		
Responsibility/Threat	4.96 (1.53)	3.71 (1.02)
Perfectionism/Certainty	5.13 (1.19)	4.15 (.99)
Importance/Control of Thoughts	4.51 (1.50)	3.00 (1.05)
ReSQ		
Source	2.62 (.93)	1.92 (.84)
Trust	3.20 (.94)	2.66 (.76)
Intensity	2.44 (.86)	1.74 (.71)
Carefulness	3.27 (.92)	2.89 (.89)
STAXI	2.46 (.71)	2.17 (.55)
GI	3.31 (.33)	3.06 (.36)

*OCI-R* Obsessive Compulsive Inventory-Revised Total Score, *OBQ* Obsessive Beliefs Questionnaire, *ReSQ* Reassurance Seeking Questionnaire, *STAXI* State and Trait Anger Expression Inventory, *GI* Guilt Inventory

### Associations between reassurance seeking and obsessive-compulsive symptoms

First, regression analyses were conducted to examine whether OCI-R total score significantly predicts the four scales of ReSQ; namely, Source, Trust, Intensity, and Carefulness. The results of the regression analyses with each scale of the ReSQ as dependent variables, and OCI-R total score as independent variable indicated that OCI-R total score was the significant predictor only for the Carefulness scale [*F*(1, 52) = 20.27, *p* < .001]. This result indicated that the OC symptom severity significantly predicted the OCD patients’ carefulness during reassurance seeking process (*β* = .53, *p* < .001) (see Table 2).

**Table 2:** Prediction of reassurance seeking on the basis of OC symptoms

Independent Variables	Dependent Variables	β	t	*R*^2^	df	*F*
OCI-R	Source	.24	1.76	.06	1, 52	3.08
	Trust	.05	.34	.002	1, 52	.11
	Intensity	.06	.42	.004	1, 52	.18
	Carefulness	.53	4.5	.53	1, 52	20.27***
Checking	Source	.18	1.15	.09	6, 52	.83
	Trust	.03	.17	.18	6, 52	1.68
	Intensity	.11	.74	.19	6, 52	1.81
	Carefulness	.13	.90	.30	6, 52	3.28
Neutralization	Source	−.08	−.36	.09	6, 52	.83
	Trust	−.46	−2.25	.18	6, 52	1.68*
	Intensity	−.37	−1.81	.19	6, 52	1.81
	Carefulness	.23	1.23	.30	6, 52	3.28
Hoarding	Source	.04	.22	.09	6, 52	.83
	Trust	.02	.11	.18	6, 52	1.68
	Intensity	.17	.99	.19	6, 52	1.81
	Carefulness	−.01	−.08	.30	6, 52	3.28
Obsessing	Source	.20	1.27	.09	6, 52	.83
	Trust	.35	2.35	.18	6, 52	1.68*
	Intensity	.40	2.74	.19	6, 52	1.81**
	Carefulness	.05	.36	.30	6, 52	3.28
Washing	Source	.03	.20	.09	6, 52	.83
	Trust	.08	.51	.18	6, 52	1.68
	Intensity	−.09	−.54	.19	6, 52	1.81
	Carefulness	.14	.89	.30	6, 52	3.28
Ordering	Source	.03	.18	.09	6, 52	.83
	Trust	.17	.98	.18	6, 52	1.68
	Intensity	.01	.06	.19	6, 52	1.81
	Carefulness	.19	1.17	.30	6, 52	3.28

*OCI-R* Obsessive Compulsive Inventory-Revised Total Score, *Control* Control subscale of OCI-R, *Neutralization* Neutralization subscale of OCI-R, *Hoarding* Hoarding subscale of OCI-R, *Obsessing* Obsessing subscale of OCI-R, *Washing* Washing subscale of OCI-R, *Ordering* Ordering subscale of OCI-R, *Source* Source Subscale of ReSQ, *Trust* Trust Subscale of ReSQ, *Intensity* Intensity Subscale of ReSQ, *Carefulness* Carefulness Subscale of ReSQ

*p < .05, ** p < .01, ***p < .001

Then, multiple regression analyses were conducted to examine the relationship between six subscales scores of OCI-R (checking, hoarding, neutralizing, obsessing, ordering and washing) and the scales of ReSQ. The results of the multiple regression analysis with each scale of the ReSQ as dependent variables, and OCI-R subscale scores as independent variables indicated that the Obsessing subscale score significantly predicted the Trust [*F*(6, 52) = 1.68, *p* < .05] and Intensity [*F*(6, 52) = 1.81, *p* < .01] scales. The results indicated that as the obsessions of OCD patients increase, the intensity of reassurance seeking (*β* = .40, *p* < .01) and the trust to the reassurance source (*β* = .35, *p* < .05) also increase. Moreover, the Neutralizing subscale score significantly predicted the Trust scale [*F*(6, 52) = 1.68, *p* < .05]. Results indicated that when the neutralizing behaviors of the OCD patients increase, the trust to the source of reassurance decreases (*β* = −.46, *p* < .05) (see Table 2).

### Associations between reassurance seeking and negative emotions

Two multiple regression analyses were conducted to examine the relationship between the scales of ReSQ and guilt and anger, with the GI and STAXI scores as dependent variables, and scales of the ReSQ as independent variables (see Table 3). The results indicated that the Intensity subscale score significantly predicted guilt scores [*F*(4, 52) = 3.19, *p* < .05], meaning that when the intensity scores of reassurance seeking of OCD patients increase, the guiltiness scores also increase (*β* = .38, *p* < .05). Moreover, the Carefulness scores significantly predicted anger scores [*F*(4, 52) = 3.87, *p* < .01], indicated that the anger scores of OCD patients increase as the scores of carefulness during reassurance seeking increase (*β* = .53, *p* < .01).

**Table 3:** Prediction of guilt and anger on the basis of reassurance seeking

Independent Variables	Dependent Variables	β	t	*R*^2^	df	*F*
Source	Guilt	−.02	−.08	.21	4, 52	3.19*
Trust		.02	.15			
Intensity		.38*	2.03			
Carefulness		.18	1.22			
Source	Anger	−.21	−1.03	.24	4, 52	3.87**
Trust		.21	1.33			
Intensity		−.12	−.64			
Carefulness		.53**	3.65			

*Source* Source Subscale of ReSQ, *Trust* Trust Subscale of ReSQ, *Intensity* Intensity Subscale of ReSQ, *Carefulness* Carefulness Subscale of ReSQ, *Guilt* Guilt Inventory, *Anger* State and Trait Anger Expression Inventory

*p < .05, ** p < .01

### The mediator role of obsessive-compulsive symptoms in the relationship between dysfunctional beliefs and reassurance seeking

One of the aims of this study was to investigate the mediating role of OC symptoms in the relationship between dysfunctional beliefs and reassurance seeking behaviors. The proposed model suggested that beliefs such as Responsibility/Threat Estimation, Perfectionism/Certainty, and Importance/ Control of Thoughts would be related to reassurance seeking behaviors through OC symptoms. In order to test this assumption, regression analyses were performed to estimate the total, direct, and indirect effects of the independent variables (subscales of OBQ) on the outcome variables (subscales of ReSQ) through the proposed mediator (OCI-R total score).

A series of regression analysis revealed that Responsibility/Threat Estimation, Perfectionism/Certainty, and Importance/ Control of Thoughts (IVs) were significantly associated with the OCI-R total score (mediator) [(SE = .06, t = 4.68, *p* < .001), (SE = .07, t = 5.28, *p* < .001), and (SE = .06, t = 4.06, *p* < .001), respectively). Moreover, the direct effects of Responsibility/Threat Estimation, Perfectionism/Certainty and Importance/ Control of Thoughts on the Source [(SE = .09, t = 2.95, *p* < .001), (SE = .12, t = 3.06, *p* < .001), and (SE = .09, t = 2.88, *p* < .01), respectively] and Carefulness [(SE = .07, t = 3.24, *p* < .001), (SE = .10, t = 3.36, *p* < .001), and (SE = .08, t = 2.95, *p* < .001), respectively] were also significant. Among the dysfunctional beliefs, only Importance/ Control of Thoughts scale significantly predicted the Intensity scores (SE = .09, t = 2.63, *p* < .01). None of the dysfunctional beliefs significantly predicted the Trust scores (see Table 4).

**Table 4:** The effect of OBQ subscale scores on the ReSQ subscale scores through the mediation of OCI-R total score

Variables	Dependent Variables	*β*	*SE*	*t*	*R*^*2*^	*F*
R/T	Source	.27	.09	2.95***	.20	6.12**
OCI-R		−.01	.18	−.04		
R/T	Trust	−.00	.10	−.01	.00	.05
OCI-R		.06	.21	.28		
R/T	Intensity	.15	.09	1.57	.05	1.32
OCI-R		−.09	.19	−.50		
R/T	Carefulness	.25	.07	3.24***	.41	17.26***
OCI-R		.37	.16	2.33*		
P/C	Source	.38	.12	3.06***	.21	6.49*
OCI-R		−.05	.19	−.29		
P/C	Trust	.01	.14	.10	.00	.06
OCI-R		.05	.21	.21		
P/C	Intensity	.16	.13	1.24	.03	.86
OCI-R		−.08	.20	−.40		
P/C	Carefulness	.35	.10	3.36***	.42	17.81***
OCI-R		.32	.16	1.67*		
I/CT	Source	.26	.09	2.88**	.19	5.92**
OCI-R		.03	.18	.20		
I/CT	Trust	.06	.10	.55	.00	.21
OCI-R		.00	.20	.01		
I/CT	Intensity	.23	.09	2.63**	.12	3.56*
OCI-R		−.15	.17	−.91		
I/CT	Carefulness	.22	.08	2.95***	.39	16.03***
OCI-R		.42	.15	2.74**		

*R/T* Responsibility/Threat Estimation, *P/C* Perfectionism/Certainty, *I/CT* Importance/ Control of Thoughts, *OCI-R* Obsessive Compulsive Inventory-Revised Total Score, *Source* Source Subscale of ReSQ, *Trust* Trust Subscale of ReSQ, *Intensity* Intensity Subscale of ReSQ, *Carefulness* Carefulness Subscale of ReSQ

*p < .05; **p < .01; *** p < .001

When the indirect effects of the independent variables on the dependent variables through the mediator were investigated, some of the observed associations between dysfunctional beliefs and reassurance seeking were maintained by OC symptoms. The mediator role of the OCI-R total score was found to be significant between Responsibility/Threat Estimation (E = .10, SE = .05, %95 CI =0.0142–0.2345) and Carefulness, and between Importance/Control of Thoughts (E = .11, SE = .05, %95 CI =0.0286–0.2071) and Carefulness. In other words, the indirect effects of Responsibility/Threat Estimation and Importance/ Control of Thoughts on Carefulness were significant through OCI-R total score. This result indicated that as the OCD patients’ dysfunctional beliefs related to responsibility/threat and importance/control of thought increase, the OC symptoms also increase, and that increased OC symptoms were associated with elevated carefulness during reassurance seeking (see Table 5).

**Table 5:** Summary of 1000 bootstraps resamples of the indirect effects of OBQ subscale scores on the ReSQ subscale scores through OCI-R total score

Independent Variables	Mediator	Dependent Variables	Direct Effect	Indirect Effect	*95%CI*
R/T		Source	.27		.0869–.4569*
	OCI-R			−.002	−.1175–.1159
R/T		Trust	−.001		−.2091–.2074
	OCI-R			.02	−.1207–.1203
R/T		Intensity	.15		−.0408–.3319
	OCI-R			−.03	−.1801–.0770
R/T		Carefulness	.25		.0954–.4075**
	OCI-R			.10	.0142–.2345***
P/C		Source	.37		.1293–.6214**
	OCI-R			−.02	−.2261–.1214
P/C		Trust	.01		−.2641–.2931
	OCI-R			.01	−.2175–.1832
P/C		Intensity	.15		−.0961–.4069
	OCI-R			−.03	−.2440–.1158
P/C		Carefulness	.34		.1394–.5541**
	OCI-R			.12	−.0027–.2684
I/CT		Source	.26		.0798–.4456**
	OCI-R			.01	−.0922–.0969
I/CT		Trust	.06		−.1480–.2612
	OCI-R			.01	−.1249–.0932
I/CT		Intensity	.23		.0548–.4072*
	OCI-R			−.04	−.1790–.0449
I/CT		Carefulness	.22		.0734–.3854*
	OCI-R			.11	.0286–.2071***

*R/T* Responsibility/Threat Estimation, *P/C* Perfectionism/Certainty, *I/CT* Importance/ Control of Thoughts, *OCI-R* Obsessive Compulsive Inventory-Revised Total Score, *Source* Source Subscale of ReSQ, *Trust* Trust Subscale of ReSQ, *Intensity* Intensity Subscale of ReSQ, *Carefulness* Carefulness Subscale of ReSQ

*p < .05; **p < .01; *** *p* < .001

### Model testing

A SEM analysis was performed based on data from 591 non-clinical participants in order to examine how the cognitive, emotional and behavioral factors related to each other and with OC symptoms. In the proposed model, it was hypothesized that dysfunctional beliefs would increase the OC symptoms, and the increased OC symptoms would lead the individuals to engage in reassurance seeking behaviors more, and the increased reassurance seeking behaviors would be related with the feelings of guilt and anger. Model fit was examined via several common indices: *χ*^*2*^ Index; Comparative Fit Index (CFI [3];), Goodness of Fit Index, Adjusted Goodness of Fit Index [7] and the Root Mean Square Error of Approximation and its 90% confidence interval (RMSEA [6, 46];). Acceptable model fit is indicated if RMSEA < 0.08 [6]; GFI, AGFI, and CFI > 0.90 [5].

**Fig. 1:**
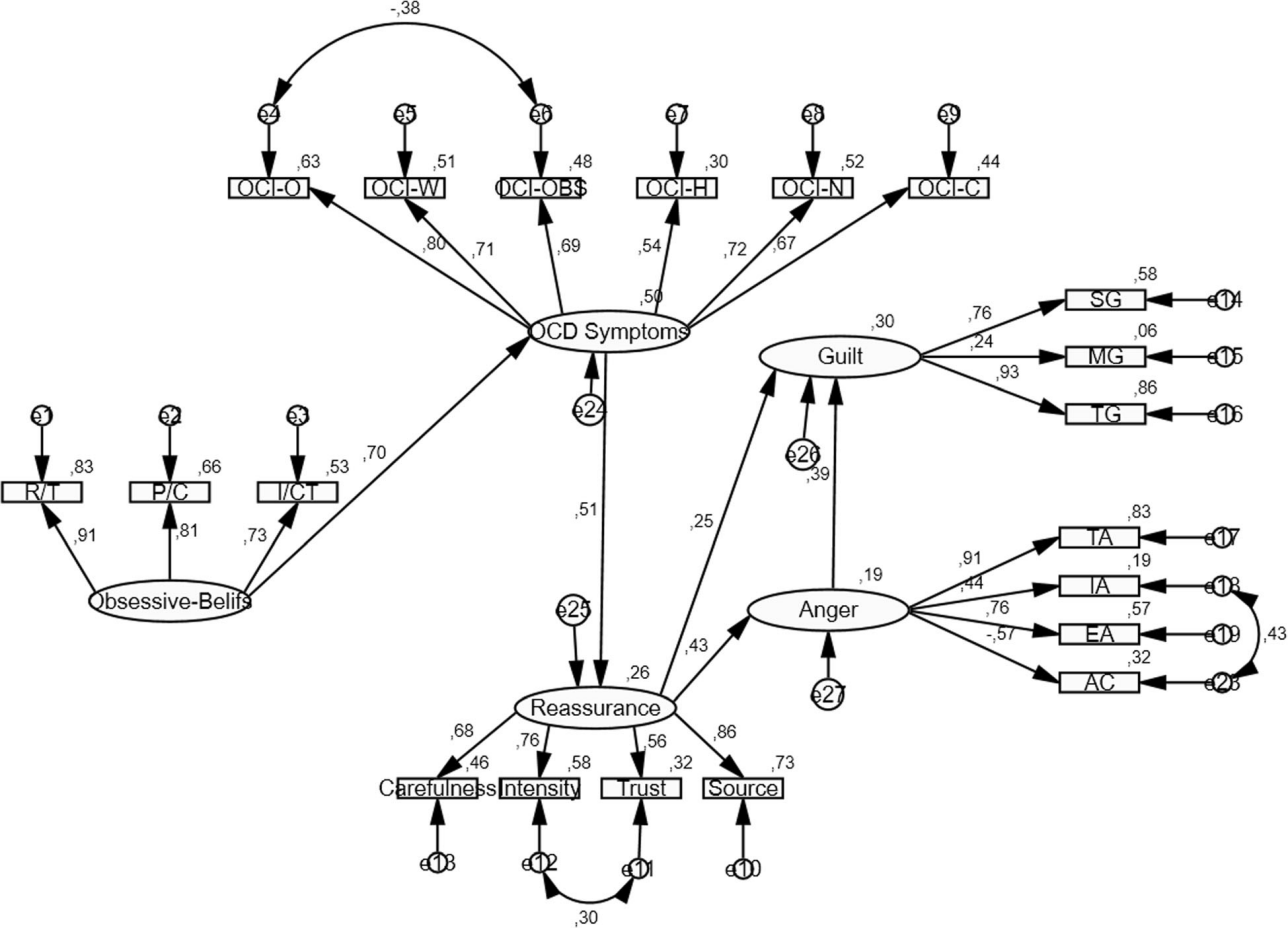
Note: R/T: Responsibility/Threat Estimation; P/C: Perfectionism/Certainty; I/CT: Importance/ Control of Thoughts; Source: Source Subscale of ReSQ; Trust: Trust Subscale of ReSQ; Intensity: Intensity Subscale of ReSQ; Carefulness: Carefulness Subscale of ReSQ; OCI-C: Control subscale of OCI-R; OCI-N: Neutralization subscale of OCI-R; OCI-H: Hoarding subscale of OCI-R; OCI-Obs: Obsessing subscale of OCI-R; OCI-W: Washing subscale of OCI-R; OCI-O: Ordering subscale of OCI-R; TA: Trait Anger; IA: Anger-in; EA: Anger-out; AC: Anger control; SG: State guilt; MG: Moral guilt; TG: Trait Guilt

The results indicated that the proposed model had inadequate fit indices (χ^2^591 = 5.56, *p* < .001, RMSEA = 0.088, AGFI = 0.82, GFI = 0.86, CFI = 0.86, NFI = 0.83, IFI = 0.86). Especially, slightly higher RMSEA (0.088) indicated that the model fit requires some modifications. Modification indices of the model suggested to add a direct path from anger to guilt (MI = 45.65), which is very compatible with OCD theoretical background. Anger and guilt are aversive emotions frequently experienced by OCD patients. For example, OCD patients usually feel distressed and guilty due to their aggressive obsessions and possible consequences of their anger [38]. Therefore, this modification was implemented and a direct path from anger to guilt was added and the model was tested. The results indicated that the fit indexes of the model improved in the expected direction (χ^2^591 = 5.22, *p* < .001, RMSEA = 0.085, AGFI = 0.83, GFI = 0.87, CFI = 0.87, NFI = 0.84, IFI = 0.87); however, further modifications should be implemented. The modification indices of the model proposed to add covariate error terms between anger-inside and anger-control in anger latent (MI = 83.25), between intensity and trust in Reassurance latent (MI = 30.29), and between ordering and obsessing in OCD symptoms latent (MI = 30.31). All suggested modifications were consistent with OCD theoretical background. The suggested modifications on adding a covariate error terms between anger-inside and anger-control were implemented because studies have shown that rather than expressing anger, individuals with OCD usually internalize anger [51], due to their inflated responsibility beliefs [30], or they try to control anger by suppressing the intrusions [49] or by using other neutralization strategies [19]. The covariate error terms between intensity of reassurance and trust in reassurance were added because it is reasonable that the intensity of the reassurance seeking increases when a person places greater trust on the reassurance source, in other words the individual repeatedly asks for reassurance from sources he/she trusts [15]. Finally, the suggested modifications on adding a covariate error terms between ordering and obsessing subscale scores were implemented. If a person has different obsessions, it is understandable that he/she might use ordering as a way of reducing the distress or anxiety. As a result, the model was corrected with these modifications and tested again. SEM results revealed that the model fit indices were improved and become acceptable (χ^2^591 = 4.18, *p* < .001, RMSEA = 0.073, AGFI = 0.86, GFI = 0.90, CFI = 0.90, NFI = 0.88, IFI = 0.90). The final standardized path coefficients in the model were shown in Fig. 1. In the structural model, dysfunctional beliefs significantly predicted OC symptoms (β = .70, *p* < .001), and explained the 50% variance in the OC symptoms. OC symptoms significantly predicted the reassurance seeking (β = .51, *p* < .001), and explained the 26% variance in reassurance seeking. Reassurance seeking also significantly predicted guilt (β = .25, *p* < .001) and anger (β = .43, *p* < .001). Guilt was also significantly associated with anger (β = .39, *p* < .001).

Finally, the significance of indirect effects of obsessive beliefs on guilt and anger was examined. A sampling with replacement, bias corrected procedure (2000 samples) was run to estimate confidence intervals (CIs) and significance levels for the indirect effects in the Model. Obsessive beliefs (via the roles of OCD symptoms, reassurance seeking, and anger) had a significant indirect effect on guilt, bias-corrected bootstrapped 95% CI [.05, .11], *p* = .004, and on anger (via the roles of OCD symptoms and reassurance seeking), bias-corrected bootstrapped 95% CI [.05, .11], *p* = .004.

## Discussion

The findings of the study supported the relationship between OC symptoms and reassurance-seeking behaviors to a certain extent. The results of the regression analysis revealed that the OCD patients may become more careful when seeking reassurance as the severity of the OC symptoms increased. These results supported the findings of previous studies that showed the associations between the severity of obsessive-compulsive symptoms and reassurance-seeking behaviors [15, 16, 45]. The Carefulness scale of the ReSQ measures the process of reassurance seeking [15]. Considering the content of the Carefulness scale, it can be asserted that OCD patients with severe symptoms put great effort when seeking reassurance. They can become more critical, carefully listen the answers they obtained and selectively attend to the reassurance provider’s face, voice tone, eagerness to give reassurance, and whether reassurance provider gives inconsistent or vague replies. Thus, the association between the increased carefulness of OCD patients and their symptom severity was consistent with the findings of previous studies [15, 16]. However, contrary to expectations, the Source, Trust, and Intensity scores of the ReSQ were not significantly associated with the OCI-R total scores. This is attributable to the small sample size of the OCD group, which can restrict the assessment of different dimensions of reassurance seeking.

The regression analysis revealed significant associations between the Obsessing and Neutralizing subscales of the OCI-R and the Trust and Intensity scales of the ReSQ. The intensity of reassurance seeking and trust to the reassurance source increased as the obsessions of the OCD patients increased. This result indicated that OCD patients may seek the same reassurance repeatedly and had the tendency to trust more to the reassurance provider when the intensity or severity of their obsessions increased. Moreover, the findings pointed out a negative relationship between neutralization and trust to the reassurance source, indicating reduced trust to the reassurance provider as the neutralization behaviors of the OCD patient increased. This can be associated with the type of neutralization strategy the OCD patient uses. For example, if the individual uses neutralization strategies other than reassurance seeking (i.e. thought suppression, distraction, and mental ritualization) or finds these strategies more reliable and useful, the trust to the source of reassurance provider can decrease. However, contrary to our expectations and previous findings [28, 32, 35, 36, 44, 45], other symptom subtypes, especially checking and washing symptoms, were not found to be significantly related to reassurance-seeking behaviors. This unexpected finding is attributable to the sample size of the OCD group. The relatively small sample size may have failed to represent the heterogeneous symptom pattern of OCD. This result can also be due to the measurement tool. Considering the revised form of OCI includes 18 items in total, some symptom types may have been omitted. Future studies should replicate the results with larger clinical samples and using other assessment tools.

The study also focuses on investigating the mediator role of OC symptoms in the relationship between dysfunctional beliefs and reassurance-seeking behaviors. It was hypothesized that dysfunctional beliefs affect reassurance-seeking behaviors through OC symptoms. As hypothesized, all dysfunctional beliefs comprising responsibility and threat estimation, perfectionism and need for certainty, and importance given to thoughts and their control significantly predicted the OC symptoms of the OCD patients. These results emphasize the importance of cognitive vulnerability factors in OCD once again, which is in line with previous studies [8, 24, 27, 31, 32, 37, 38, 40, 48]. Moreover, the dysfunctional beliefs directly associated with an increased need to seek reassurance from different sources and seek reassurance more carefully. The dysfunctional belief related to the importance of thoughts and their control also affected OCD patient’s repeated reassurance seeking. In terms of mediational effect, the results revealed that the individuals who had distorted beliefs regarding inflated responsibility, threat estimation, importance of thoughts, and control of thoughts were more likely to have OC symptoms. In turn, these OC symptoms can increase carefulness when seeking reassurance. The results of this study supported the findings of previous studies, which identified perceived responsibility and threat estimation as the major motivations to seek reassurance [16, 27, 37, 38, 40]. The underlying cognitive vulnerability that the person has the responsibility to cause/prevent an aversive event can lead the person to view the intrusive thought as a signal of threat. To diminish the anxiety caused by the intrusive thought, the person requires reassurance that he/she is not responsible or he/she takes all the necessary precautions to prevent negative outcomes or shares the perceived responsibility just by talking about the situation [27, 37, 38, 40]. Similarly, if the OCD patient believes that the presence of a thought is the signal of the thought’s significance and that the thought can/should be controlled, the dysfunctional beliefs increase the OC symptoms and the distress caused by the unwanted intrusive thoughts can lead the person to seek reassurance more carefully.

The negative emotions triggered by obsessions have been frequently reported by previous empirical studies. The present study examines the negative emotions that are related to neutralization behaviors, namely reassurance-seeking behaviors. The findings of the study further support the limited number of studies examining reassurance seeking-related emotions [15–17, 28]. The results of the regression analysis revealed that feelings of guilt increased with increasing intensity of reassurance seeking, in other words, when the OCD patients sought the same reassurance repeatedly from a reassurance source, they felt guilty. Moreover, the results suggested that OCD patients’ feelings of anger increased as they became more critical and careful during reassurance seeking. As stated before, being careful during reassurance seeking involves paying attention to how reassurance was given [15]. Therefore, the OCD participants may have been more vigilant for verbal or nonverbal signals and the inconsistent, vague, or contradictory replies of the reassurance provider may have angered the OCD patients. Consistent with these findings, in their qualitative study, Kobori, Salkovskis, Read, Lounes and Wong [16] identified some common themes in the interviews with OCD patients about reassurance seeking from other people, which included interrogating feelings to achieve a sense of certainty, ceaseless and careful effort, reluctance to seek reassurance, and interpersonal concern. The researchers reported that although individuals with OCD had a strong urge to seek reassurance, they also expressed embarrassment, fear, and guilt, as they did not want to seem mad or weak or bother the reassurance provider by persistent requests. Therefore, feelings of guilt lead some patients to secretly seek reassurance or lead to efforts of compensating for their annoying need for reassurance [16].

Lastly, all variables were investigated in a comprehensive model that can help explain “how” OC symptoms persist. The proposed model was tested using a non-clinical sample due to the small sample size of the OCD group. The analysis of the model test revealed mostly similar results to those obtained for the clinical sample. In the model, as hypothesized, the dysfunctional beliefs had a significant effect on the severity of the OC symptoms. Increased OC symptoms were associated with seeking reassurance more frequently from a range of sources, trusting these sources more, seeking reassurance more intensely, and becoming more careful when seeking reassurance. Moreover, increased reassurance-seeking behaviors affected the increase in feelings of guilt and anger.

To the best of our knowledge, no previous studies have tested the mediational role of OC symptoms and reassurance-seeking behaviors in the relationship between dysfunctional beliefs and negative emotions. Consistent with the cognitive-behavioral theories regarding OCD [8, 24, 31, 32, 37, 38], once again, our findings showed the importance of dysfunctional beliefs as cognitive vulnerability factors. Inflated responsibility, threat estimation, perfectionism, need for certainty, and importance of thoughts and their control had significant effects on reassurance seeking through OC symptoms. These distorted beliefs make the person more prone to evaluate an intrusive thought as a signal of threat and the person seek reassurance with accompanying feelings of guilt and anger to decrease the distress caused by intrusions. However, excessive reassurance-seeking behaviors contribute to the maintenance of the OC symptoms in the long term, similar to other neutralizing behaviors and safety strategies.

The clinical implication of the study is that, during therapy, the professionals should not disregard the importance of questioning whether the patient uses reassurance-seeking behaviors as a safety strategy, which sources are frequently and intensely used, and what are the processes and consequences of reassurance-seeking behaviors. One of the important contributions of the study was investigating the mediator role of reassurance-seeking behaviors in the relationship between OC symptoms and negative emotions. The findings pointed out that aversive emotions were possibly not only related to the content of the obsessions but also to other factors such as reassurance-seeking behaviors. Therefore, the sources of negative emotions should be separately examined and handled during the intervention process. The anger and guilt feelings related to reassurance-seeking behaviors should be questioned and dealt with during the therapy process. Explaining the role of seeking and providing reassurance in the persistence of the symptoms and informing the patient about the possible emotions raised by reassurance seeking and their effects both on the patient and on the environment, i.e. family members, will help the treatment of OCD. In conclusion, the reassurance-seeking behaviors should be questioned and targeted during therapy both to evaluate the individual with OCD more comprehensively and improve the outcome of the therapy.

There are some limitations to the present study. Firstly, the study had a non-experimental design and, thus, the results of the analysis were correlational in nature. The other limitation is the sample size of the OCD group. The results should be replicated with larger clinical samples that can represent the heterogeneous symptom subtypes of OCD. In addition, the model should be tested with a clinical sample. Moreover, the DSM-IV criteria were used for the diagnosis of the patients. Furthermore, OCD patients with comorbid diagnosis were excluded from the study to eliminate confounding factors and investigate the specificity of the proposed associations to OCD. This can affect the generalizability of the findings because empirical studies and clinical experiences have indicated high comorbidity rates of OCD with depression and anxiety disorders. Another limitation is that the participants in the non-clinical sample were not screened through a structured instrument (SCID-I). Therefore, the exclusion of the participants with a psychiatric disorder was based only on the self-reports of the participants. Finally, this study did not investigate the role of gender in reassurance-seeking behaviors. Thus, future studies should also investigate the effects of gender differences on OCD patients’ reassurance-seeking behaviors and related emotions.

## Conclusions

This study supports the associations between reassurance-seeking behaviors, dysfunctional beliefs, negative emotions, and obsessive-compulsive symptoms. Therefore, similar to other safety strategies, the maintaining role of reassurance-seeking behaviors of the OCD patients should not be disregarded and the interpersonal aspect of reassurance-seeking behaviors and accompanying aversive emotions should be handled to achieve better therapy outcomes.

## Data Availability

The datasets used and/or analyzed during the current study are available from the corresponding author on reasonable request.

## Abbreviations

AGFI: Adjusted Goodness of Fit Index
AMOS: Analysis of Moment Structures
CFI: Comparative Fit Index
CI: Confidence interval
GFI: Goodness of Fit Index
GI: Guilt Inventory
IFI: Incremental Fit Index
NFI: Normed Fit Index
OBQ: Obsessive Beliefs Questionnaire
OC: Obsessive Compulsive
OCCWG: Obsessive Compulsive Cognitions Working Group
OCD: Obsessive Compulsive Disorder
OCI-R: Obsessive-Compulsive Inventory-Revised Form
ReSQ: Reassurance Seeking Questionnaire
RMSEA: Root Mean Square Error of Approximation
SCID-I: Structured Clinical Interview for DSM-IV Axis I Disorders
SEM: Structural Equation Modeling
STAXI: State and Trait Anger Expression Inventory
